# Effects of a virtual reality-based motivational reinforcement + desensitization intervention program on psychological craving and addiction memory in female MA-dependent young adults

**DOI:** 10.3389/fpsyt.2023.1114878

**Published:** 2023-07-25

**Authors:** Xihui Ji, Yuyao Tang, Lushi Jing, Li Zhou, Binbin Wu, Yong Deng, Sijin Zhou, Yangyan Yang

**Affiliations:** ^1^School of Psychology, Chengdu Medical College, Chengdu, China; ^2^Sichuan Women’s Compulsory Isolation Drug Treatment Center, Deyang, Sichuan Province, China

**Keywords:** MA-dependent, VR, motivational reinforcement, desensitization, psychological craving, addictive memory

## Abstract

**Objectives:**

The aim of this study was to explore the effects of a virtual reality (VR)-based motivational reinforcement + desensitization intervention program on psychological craving and addiction memory in female methamphetamine (MA)-dependent young adults.

**Methods:**

We recruited 60 female MA-dependent young adults in a compulsory isolation drug rehabilitation facility in Sichuan Province, and randomly assigned them to intervention (mean age = 23.24 ± 2.06) and control groups (mean age = 23.33 ± 2.09). The intervention group received a VR-based motivational enhancement + desensitization intervention (total of eight sessions over a 4-week period), while the control group received regular detoxification management during the same period. Assessments were conducted before, immediately after, and 1 month after the intervention, with a visual analogue scale (VAS) being used to assess subjective craving, electronic sphygmomanometer employed to measure physiological parameters, and the Addiction Memory Intensity Scale (AMIS) applied to assess addiction memory intensity.

**Results:**

Generalized estimating equation analysis showed significant main effects of group on changes in heart rate difference, systolic blood pressure difference, VAS and AMIS scores (all *p* < 0.01), and a significant time main effect on changes in diastolic blood pressure difference, VAS and AMIS scores (all *p* < 0.01), and a significant group × time interaction effect on changes in the difference values of three physiological parameters, VAS and AMIS scores (*p* < 0.01 or *p* < 0.05). After the intervention, the differences in three physiological parameters, and the VAS and AMIS scores, were significantly lower in the intervention than in the control group (all *p* < 0.05), and the difference between the two groups remained significant 1 month after the end of the intervention (both *p* < 0.01). VAS scores, heart rate difference, and diastolic blood pressure difference in the intervention group were significantly lower than baseline scores, both at the end of the intervention and 1 month thereafter (all *p* < 0.01); the systolic blood pressure difference in the intervention group was significantly lower at the end of the intervention than at baseline (*p* < 0.05); AMIS scores in the intervention group were significantly lower than the baseline scores 1 month after the end of the intervention (*p* < 0.01).

**Conclusion:**

Our VR-based motivational reinforcement + desensitization intervention program can effectively reduce psychological craving and physiological reactivity for drugs, and the intensity of addictive memories in female MA-dependent young adults, even after 1 month.

## 1. Introduction

Methamphetamine (MA) has high addiction and dependence potential, and is neurotoxic (1). Prolonged use can lead to adaptive changes in the nervous system and brain, resulting in strong psychological cravings and subsequent relapse. Relapse has always been the main focus and challenge for treating MA dependence. Motivation is closely related to treatment adherence and outcomes (2), but MA-dependent individuals are less motivated than individuals dependent on more traditional drugs of abuse (3). Strengthening motivation is a prerequisite for treatment. The persistence of addiction memories is key to the psychological craving experienced by addicts, and the behaviors that lead to relapse; interventions that target addiction memories have treatment efficacy. Compared with adults, the brains of young adults are more susceptible to psychological craving stimulated by addictive drugs (4), and adolescence is a critical period characterized by increased brain plasticity (5). During this period, it is important to strengthen the motivation of MA-dependent individuals and intervene to address their psychological craving and addiction memory to prevent relapse.

Intensive motivational treatment is a widely used approach in which the therapist employs certain strategies to help patients build and enhance their motivation and goals, and thus address their abusive behavior. Motivational interviewing is the main form of motivational intensive treatment, and can effectively improve the motivation and treatment adherence of MA-dependent individuals (6). According to memory reconsolidation theory, by interfering with the reconsolidation process of the original addiction memory, it is possible to modify or alter it, reduce the craving response after memory arousal, and decrease drug use behavior (7). A growing number of researchers are applying memory reconsolidation theory to addiction interventions. Research has shown that interventions that activate addiction memories and intervene in memory reconsolidation can be effective for reducing cravings and substance use behaviors in addicted individuals (8, 9). Virtual reality (VR) is based on computer technology that generates a three-dimensional environment with high similarity to the real environment in terms of sight, sound, and tactile sensations; the equipment allows people to fully interact with the environment, and generates immersive feelings and experiences (10). Due to the high ecological validity of VR technology, it is superior to traditional stimuli, such as pictures and videos, in terms of activating addictive memories and inducing craving (11). However, few studies have examined the efficacy of interventions that combine VR with memory reconsolidation intervention techniques, and there is even less evidence for the clinical efficacy of integrating motivational reinforcement into this approach.

In this study, we propose the use of motivational interviewing to enhance the motivation of MA-dependent adolescent females to detoxify, and use VR technology to create MA-related scenarios to activate their addiction memories and desensitize them during memory reconsolidation. We also evaluate the effects of this new method on the strength of addiction memories and psychological craving of female MA-dependent young adults.

## 2. Methods

### 2.1. Study design and participants

This was a randomized, controlled, single-blind, priority study. Sample size was calculated through the G*Power software, *α* = 0.05, 1 - β = 0.95, the number of levels of the between-group variable is 2, the number of repeated measurements is 3. In order to achieve a medium effect size, the calculated sample size required is 22 people per group. Due to the long study period, a 5% dropout rate was assumed based on the results of previous literature (12). Therefore, we aimed to recruit 30 people for each group.

We recruited 60 MA-dependent female young adults who met the inclusion criteria and exclusion criteria from a compulsory drug rehabilitation center in Sichuan Province, from June 10 to July 10, 2022. The inclusion criteria were as follows: (1) mainly use MA-type drugs; (2) meet the ICD-10 (International Classification of Diseases) diagnostic criteria for amphetamine-type drug dependence; (3) no brain trauma or history of mental illness; (4) normal vision (no color blindness or weak color vision); (5) aged 18–25 years; and (6) fully understand the study content. Exclusion criteria: (1) presence of drug use (e.g., heroin, cocaine); (2) brain injury and coma of more than 30 min; (3) history of mental illness or family history of mental illness; (4) visual acuity or corrected visual acuity of less than 1.0; (5) illiteracy.

After completing the recruitment process, we randomly assigned the eligible MA-dependent young adults to intervention and control groups (30 participants per group). Block randomization was used, with blocks of random length and random changes in block sizes (4, 6, or 8). A random number table was generated by the principal investigator and handed to a research assistant blinded to information relevant to the experiment. The assistant informed each participant of their group assignment. A pretest assessment of psychological craving, physiological parameters, and addiction memory intensity, using a visual analogue scale (VAS), electronic sphygmomanometer, and the Addiction Memory Intensity Scale (AMIS), was then administered to all participants. In the intervention phase, the intervention group received a VR-based motivational reinforcement + desensitization intervention, and the control group received regular detoxification management. We assessed the intensity of addiction memory, physiological parameters, and psychological craving in both groups, immediately and 1 month after the end of the intervention.

This study was approved by the Medical Ethics Committee of Chengdu Medical College (approval number: 2022NO.23). All subjects voluntarily participated and signed the informed consent form. Subjects who did not want to continue to participate in the study for any reason could withdraw at any time.

### 2.2. Instruments

#### 2.2.1. Virtual reality tools

**Figure 1:**
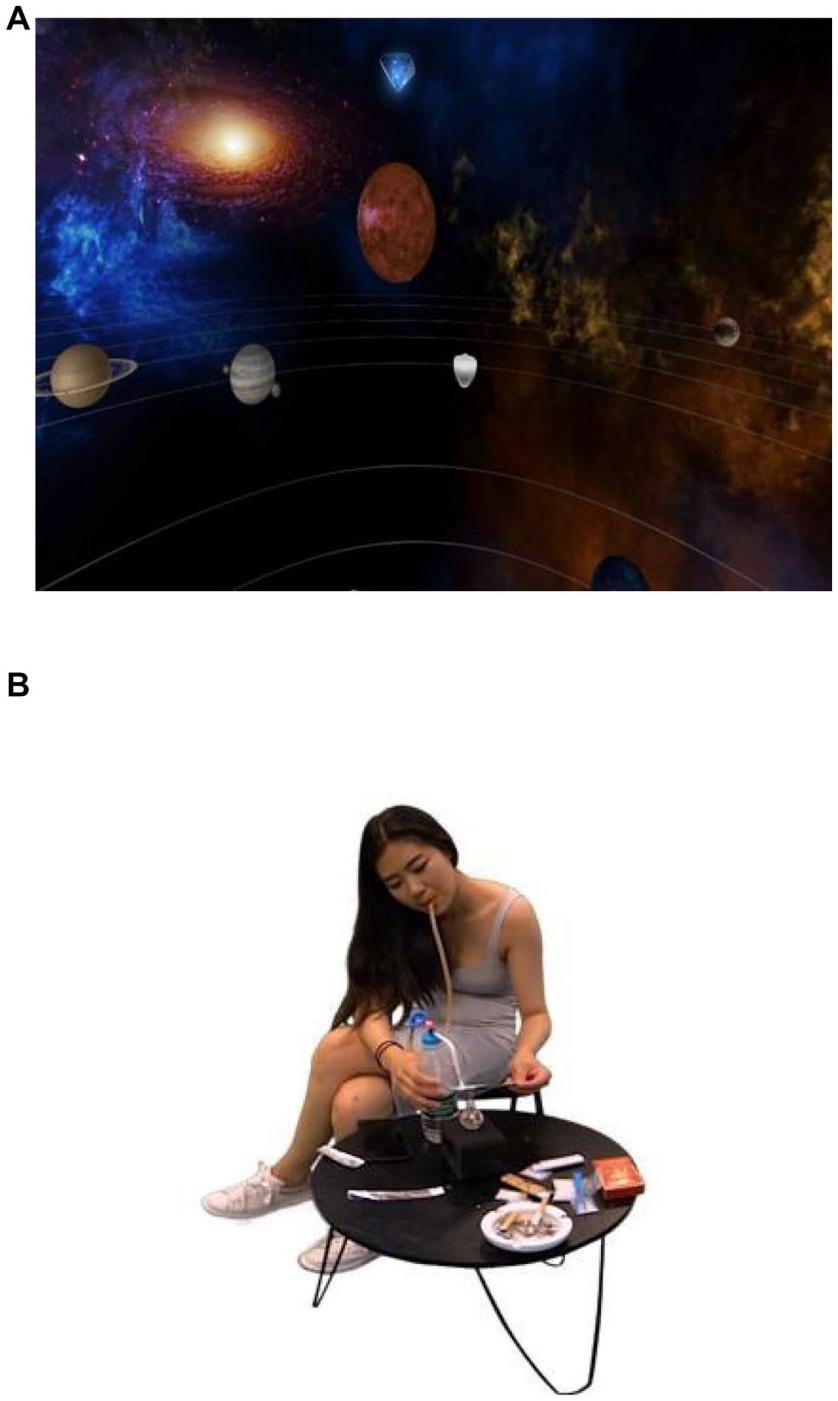
VR scenes.

This study used the PICO G2 device to create a VR environment. PICO G2 is a VR head-mounted display developed by Bird See Technology Co. (Beijing, China). The device can create VR scenes, including neutral and MA-related scenes. Neutral scenes: starry sky, grass, etc.; MA-related scenes: the whole process of a woman taking MA (Figure 1).

#### 2.2.2. General information questionnaire

We self-designed a general information questionnaire to collect demographic information, including age, education level, marital status, and length and amount of drug use.

#### 2.2.3. Visual analogue scale

A VAS (13) was used to evaluate the participants’ subjective psychological craving for drugs. The VAS was initially used clinically to rate pain intensity, and has since been widely used in the field of addiction (14, 15). It has good validity for assessing subjective craving, and was the main outcome measure of this study. The VAS used in this study was a 10-cm line [left endpoint (0), “no craving at all”; right endpoint (100), “very strong craving”]. Subjects placed a mark on the VAS according to their degree of subjective drug craving, and the distance between the marked point and the left endpoint was taken as the craving score. Higher scores indicate higher subjective craving.

#### 2.2.4. Instruments for measuring physiological indicators

Heart rate and blood pressure reflect an individual’s altered emotional state and may indirectly reflect the participant’s state of craving and addictive memory activation. Heart rate and blood pressure are the primary indicators of cue reactivity and are often considered objective measures of anxiety and craving responses (16). When patients with SUDs are exposed to drug-related cues, their heart rate and blood pressure may increase (17). In this study, heart rate and blood pressure were used as physiological indicators, and the differences in physiological indicators (physiological parameters after the VR experience - physiological parameters before the VR experience), was used as the primary outcome variable. Heart rate and blood pressure were measured using the CK-W356 electronic sphygmomanometer (Zhuochen). During the measurements, the subject’s left hand is placed palm up, and the sphygmomanometer is wrapped around the inside of the left wrist, fixed at a distance of 10–15 mm between the base of the palm and the wrist, and kept at the same height as the heart. The subject is told to stay relaxed during the measurement, press the switch, and wait for 20s for the blood pressure and pulse rate recording to begin.

#### 2.2.5. Addiction memory intensity scale

The AMIS was used to assess the addiction memory intensity of the study participants, and the total score of the scale and its dimensional scores were used as secondary outcomes. The AMIS was developed by Chen et al., and mainly measures visual clarity among other sensory aspects of addictive memories (18). The nine AMIS items are scored using a Likert 5-point scale ranging from 1 (“not at all”) to 5 (“completely”). Higher scores indicate more intense addictive memories. The Cronbach’s α was 0.93 in this study.

### 2.3. Procedures

#### 2.3.1. Experimental procedure

The experiment consisted of preparation, assessment and intervention phases. During the preparation stage, the experimenter briefly discussed the study purpose and procedure, as well as the concepts of psychological addiction and addiction memory, and the principles of the treatment, so that the participants had a degree of understanding of the treatment process. In addition, the experimenter creates an inclusive and relaxed atmosphere, proactively acquires basic information about the participants. And discusses the participants’ experiences of growing up with addiction. The goal is to build relationships and stimulate motivation for recovery. Then, the participants were instructed to sign the informed consent form and complete the general information questionnaire. During the assessment phase, the participants were assessed for craving, physiological parameters, and addiction memory intensity using the VAS, electronic sphygmomanometer, and AMIS, which were administered before, immediately after, and 1 month after the intervention. For the assessment, participants wore VR headsets that presented neutral, MA-related, and neutral scenes in sequence, for a total of 10 min. Before and after presenting the scenes. The participants’ heart rate and blood pressure were measured (as physiological indicators). After each scene was presented, the experimenter guided the participants to complete the VAS and AMIS, in that order. In addition, after using the VR equipment, the experimenter conducts a qualitative interview with each participant to assess the VR experience; those who are not comfortable with it can withdraw from the experiment at any time. The interview noted any instances of a sense of vertigo, vomiting, or sense of immersion. During the intervention phase, the intervention group received VR-based motivational reinforcement + desensitization intervention (total of eight sessions for 4 weeks) in addition to routine drug rehabilitation management; the control group received routine drug rehabilitation management during the same period. During the intervention period, the routine drug rehabilitation management consisted of no contact with drugs and some simple manual work. The intervention and evaluation stages were implemented by professionally trained psychology graduate students.

#### 2.3.2. Interventions

The intervention primarily followed a group therapy format, although one-on-one motivational interviews of the participants were conducted by the therapist prior to the start of the group therapy. The group therapy was divided into two stages. The first stage was a motivational reinforcement phase, comprising two sessions completed within 1 week and a single group session 60 min in length. The second stage was VR-based desensitization therapy (two sessions per week; six sessions in total completed in 3 weeks and a single group session 60 min in length). The specific content of each stage of the intervention is shown in Table 1.

**Table 1:** Virtual reality-based motivation reinforcement-desensitization therapy.

Stage	Aims	Main contents
**One-on-one motivational interviews:**	**One-on-one motivational interviews:** 1. Building therapeutic relationships and forming therapeutic alliances. 2. Motivating participants to detoxify.	**One-on-one motivational interviews:** 1. The therapist conducts individualised psychological interviews based on each participant’s personal attributes to explore the participant’s motivation and available inner resources to activate motivation for recovery.
**Group therapy: motivational reinforcement phase:**	**1st group meeting:** 1. Forming a group, establishing group norms and familiarising each group member with each other. 2. Clarifying group aims and motivations for group participation. **2nd group meeting:** 1. Becoming aware of the causes and effects of drug addiction and exploring the expectations of recovery and the meaning of life. 2. Reinforcing motivation for detoxification.	**1st group meeting:** 1. The therapist forms the group and clarifies group norms (including: specific timing of group meetings, active expression of ideas, respectful listening, privacy and confidentiality, etc.). 2. Group members introduce themselves, giving their name and a story about their name or what their family expects of them after their name. 3. Each group member talks about their motivation for participating in the group and the therapist leads the group in setting goals together. **2nd group meeting:** 1. The therapist leads the group to review the previous group summary. 2. Group members discuss the effects of drug use on themselves in the group. 3. The therapist leads the group in a discussion about the meaning of life and the relationship between drug use and the meaning of life. 4. Group members write down their expectations of recovery and hand them to the therapist for safekeeping. 5. Assignment: Think about “Are there other possibilities for my life if I do not take drugs?”
**Group therapy: VR-based desensitization treatment**	**3rd ~ 7th group meeting:** 1. Activating addictive memorie and inducing psychological craving through exposure to MA-related scenarios in a virtual reality environment. 2. Reducing addictive memorie and psychological craving through relaxation exercises within an effective time window. 3. Sharing of therapeutic experiences and personal insights in a group to reinforce the effects of therapy and gain group support. **8th Group Meeting:** 1 ~ 3.Same as the previous five group meetings. 4. Summarising the gains and say goodbye to the group.	**3rd ~ 7th group meeting:** 1. The therapist leads the group in reviewing the last group summary and discussing reflections on the last assignment. 2. The group members entered a virtual MA-related scene through a VR headset and were exposed to it for 3–5 min. 3. After the group members took off their helmets, the therapist played relaxing music and guided them in the performance of relaxation exercises. 4. The group members shared their experience of treatment and reported their subjective psychological craving. 5. The therapist summarized the session, and guided the group members in terms of reviewing the treatment process and applying relaxation skills. 6. Assignment: Think about “What methods can you use to reduce your craving for drugs when and after a tip is exposed?” “How should you respond to drug-related clues in the future?” **8th Group Meeting:** 1 ~ 5.Same as the previous five group meetings. 6. The group members share what they have learned and how they have grown personally after participating in the eight groups and how they feel about the other group members. The therapist guides the group to say goodbye to the group.

### 2.4. Quality control

Design phase: The experimental protocol was designed on the basis of the first author’s extensive reading of the literature, training in psychological techniques, practice of psychological counselling and psychological interventions in drug treatment and in-depth exchanges with peers. The detailed experimental plan was determined after several rounds of revision by the members of the project team.

Implementation phase: The research leader, who is the corresponding author of this paper, provides special training to the psychology postgraduate students, modelling the intervention scenarios, anticipating possible contingencies and formulating practical and effective countermeasures. The entire intervention and evaluation process is carried out by the psychology postgraduate students who have been trained according to the experimental plan.

### 2.5. Statistics

All of the data were analyzed using IBM SPSS.22.0 software (IBM Corp., Armonk, NY, United States). Descriptive statistics are presented as means ± standard deviations, frequencies, or percentages. An independent sample t-test or Chi-square test was used to compare the demographic characteristics between the intervention and control groups. Using paired samples t-tests to compare differences in physiological indicators before and after entering meth-related VR scenarios for all study participants. Taking psychological craving, differences in physiological indicators (physiological parameters after the VR experience - physiological parameters before the VR experience), and addictive memory strength as the outcome measures, the generalized estimating equation (GEE) was used to analyze the intervention effect. The significance level was set at *p* < 0.05, and the marginal significance level was set at *p* < 0.1. GEE is a semi-parametric statistical method based on likelihood estimation often used for analyzing repeated-measures data. It is applicable to outcome variables that are not normally distributed (19).

## 3. Results

### 3.1. Demographic data

**Figure 2:**
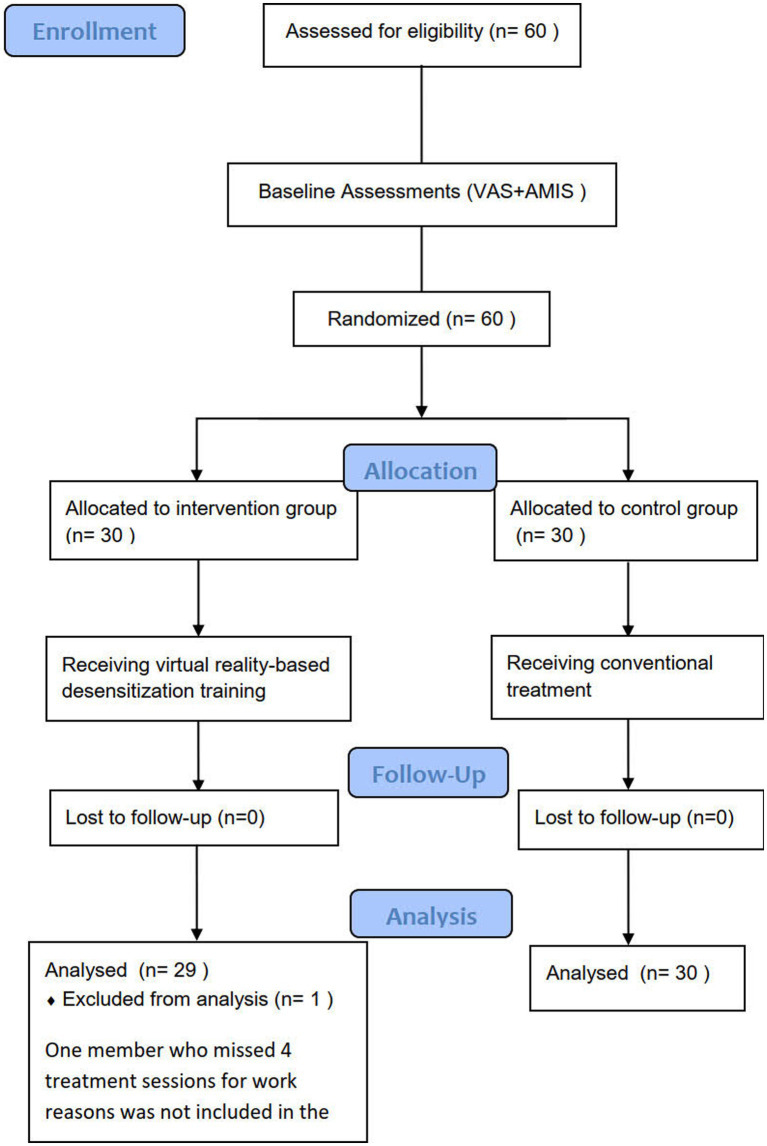
Flow diagram.

The recruitment process is shown in Figure 2. In total, 60 MA-dependent young adults participated in this study, and all of them completed measurements before, immediately after, and 1 month after the intervention. During the intervention, one member of the intervention group missed four treatment sessions for work reasons and was not included in the analysis. Ultimately, 29 and 30 valid samples were obtained for the intervention and control groups, respectively.

There was no significant difference in demographic characteristics between the intervention and control groups (Table 2). All of the participants were female. The mean age was 23.24 ± 2.06 in the intervention group and 23.33 ± 2.09 in the control group. Among all subjects, 36 (61%) lived in cities, 15 (25.4%) lived in towns, and 8 (13.6%) lived in rural areas. In total, 45 (76.2%) subjects had a primary or junior high school education, and 14 (23.8%) had a senior high school education or above. In total, 33 (55.9%) subjects were the only child, while 26 (44.1%) were not the only child in the family. Furthermore, 32 (54.2%) subjects were from single-parent families and 27 (45.8%) were from non-single-parent families. There were 41 (69.5%) unmarried subjects, 12 (20.3%) married subjects, and 6 (10.2%) divorced subjects. Twenty-three participants (38.9%) were employed before admission, and 36 (61.1%) were unemployed. In all participants, the shortest duration of drug use was 1 year and the longest was 10 years. The average duration of drug use was 4.90 ± 2.65 years in the intervention group and 4.70 ± 1.71 years in the control group, and the average amount of drug use per occasion was 0.70 ± 0.42 g in the intervention group and 0.56 ± 0.38 g in the control group.

**Table 2:** Participants’socio-demographic characteristics at baseline.

Variables		Intervention group (*n* = 29)	Control group (*n* = 30)	χ^2^/t	*p*
Age(Years) ±*s*		23.24 ± 2.06	23.33 ± 2.09	−0.170	0.866
Habitation *n* (%)	City	15 (25.4)	21 (35.6)	4.751	0.093
	Town	11 (18.6)	4 (6.8)		
	Country	3 (5.1)	5 (8.5)		
Education *n* (%)	Primary or junior high school	24 (40.7)	21 (35.5)	1.326	0.249
	High school or above	5 (8.5)	9 (15.3)		
Only children *n* (%)	Yes	18 (30.5)	15 (25.4)	0.871	0.351
	No	11 (18.6)	15 (25.4)		
From single-parent family *n* (%)	Yes	16 (27.1)	16 (27.1)	0.020	0.887
	No	13 (22.0)	14 (23.7)		
Martial status *n* (%)	Unmarried	23 (38.9)	18 (30.5)	5.166	0.056
	Married	3 (5.1)	9 (15.3)		
	Divorced	3 (5.1)	3 (5.1)		
Working condition *n* (%)	Employed	14 (23.7)	9 (15.3)	2.071	0.150
	Unemployed	15 (25.4)	21 (35.5)		
Duration of drug abuse (Years) ± *s*		4.90 ± 2.65	4.70 ± 1.71	0.340	0.735
Amount of drug use (per occasion) (g) ± *s*		0.70 ± 0.42	0.56 ± 0.38	1.327	0.190

### 3.2. Effects of VR environments on physiological indicators

Comparing the physiological indicators of all subjects before and after entering the MA-related VR scenes, a significant increase in heart rate and blood pressure was found (all *p* < 0.0001). The results are shown in Table 3.

**Table 3:** Comparison of physiological indexes before and after entering the MA-related VR scene.

Variables	before entering the MA-related VR scene	after entering the MA-related VR scene	*t*	*p*
HRD	70.95 ± 10.12	76.16 ± 9.32	6.480	0.000^**^
DBPD	102.07 ± 11.54	110.90 ± 10.40	6.068	0.000^**^
DBPD	70.41 ± 10.04	77.83 ± 11.81	4.922	0.000^**^

HRD, heart rate difference; DBPD, diastolic blood pressure difference; SBPD, systolic blood pressure difference. ^*^p < 0.05, ^**^p < 0.01.

### 3.3. Treatment effects

The effect of the intervention was analyzed by a 2 (group: intervention and control) × 3 (test time: baseline, immediately post-test, and 1-month post-test) GEE, and the results are shown in Tables 4, 5.

**Table 4:** VAS score and the difference value in physiological parameters, and differences between groups and times according to the Generalized estimating equation (GEE) analysis.

		Intervention group (*n* = 29)				Control group (*n* = 30)					
Variables	Time	±*s*	Difference between times (95%CI)	Wald*χ*^2^	*p*	±*s*	Difference between times (95%CI)	Wald*χ*^2^	*p*	Difference between groups (95%CI)	*p*
VAS	Baseline	36.72 ± 3.13				30.17 ± 4.14				6.56 (−3.62,26.74)	0.222
	Immediately post-test	7.59 ± 1.65	29.14 (22.65,35.62)	77.582	0.000^**^	27.67 ± 3.99	2.5 (−5.51,10.51)	0.38	0.54	−20.08 (−28.54,11.62)	0.000^**^
	1-month post-test	10.17 ± 2.42	26.55 (20.60.32.50)	76.452	0.000^**^	27.67 ± 3.58	2.5 (−4.52,9.52)	0.49	0.49	−17.49 (−25.96,-9.02)	0.000^**^
HRD	Baseline	5.07 ± 1.30				5.23 ± 1.04				−0.16 (−3.43,3.10)	0.921
	Immediately post-test	1.21 ± 0.37	3.86 (1.71,6.01)	12.402	0.000^**^	6.47 ± 0.92	−1.23 (−2.00,-0.47)	9.97	0.002^**^	−5.26 (−7.21,-3.31)	0.000^**^
	1-month post-test	1.45 ± 0.36	3.62 (1.34,5.90)	9.715	0.002^**^	6.36 ± 0.76	−1.13 (−2.53,0.26)	2.53	0.11	−4.92 (−6.56,-3.27)	0.000^**^
DBPD	Baseline	9.17 ± 2.32				8.53 ± 1.66				0.64 (−4.95,6.22)	0.823
	Immediately post-test	2.66 ± 0.85	6.52 (2.31,10.73)	9.199	0.002^**^	8.20 ± 1.49	0.33 (−0.49,1.16)	0.63	0.43	−5.54 (−8.92,-2.17)	0.001^**^
	1-month post-test	3.17 ± 0.89	6.00 (1.76,10.24)	7.692	0.006^**^	8.30 ± 1.68	0.23 (−1.62,2.09)	0.06	0.81	−5.13 (−8.86,-1.40)	0.007^**^
SBPD	Baseline	7.21 ± 2.44				7.66 ± 1.66				−0.46 (−6.25,5.33)	0.876
	Immediately post-test	2.17 ± 0.61	5.03 (0.63,9.44)	5.026	0.025^*^	9.50 ± 2.44	−1.83 (−5.49,1.82)	0.97	0.32	−7.33 (−12.25,-2.40)	0.004^**^
	1-month post-test	2.31 ± 1.10	4.90 (−0.04,9.84)	3.776	0.052	6.83 ± 1.53	0.83 (−1.64,3.31)	0.44	0.51	−4.52 (0.83,8.22)	0.016^*^

VAS, visual analogue scale; HRD, heart rate difference; DBPD, diastolic blood pressure difference; SBPD, systolic blood pressure difference. CI, confidence interval, ^*^p < 0.05, ^**^p < 0.01.

**Table 5:** AMIS, VC, and OSA scores and differences between groups and times according to the Generalized estimating equation (GEE) analysis.

		Intervention group (*n* = 29)				Control group (*n* = 30)					
Variables	Time	±*s*	Difference between times (95%CI)	Wald*χ*^2^	*p*	±*s*	Difference between times (95%CI)	Wald*χ*^2^	*p*	Difference between groups (95%CI)	*p*
AMIS	Baseline	3.02 ± 0.16				3.13 ± 0.12				−0.11 (−0.50,0.28)	0.586
	Immediately post-test	2.81 ± 0.14	0.21 (−0.10,0.52)	1.722	0.19	3.25 ± 0.10	−0.12 (−0.33,0.07)	1.48	0.22	−0.45 (−0.79,-0.10)	0.037^*^
	1-month post-test	2.55 ± 0.11	0.47 (0.23,0.70)	14.592	0.000^**^	3.22 ± 0.12	−0.09 (−0.27,0.08)	1.132	0.29	−0.67 (−0.99,-0.35)	0.000^**^
VC	Baseline	3.17 ± 0.17				3.26 ± 0.12				−0.09 (−0.50,0.32)	0.678
	Immediately post-test	2.97 ± 0.16	0.20 (−0.15,0.56)	1.259	0.26	3.40 ± 0.10	−0.14 (−0.34,0.06)	1.833	0.18	−0.43 (−0.79,-0.68)	0.027^*^
	1-month post-test	2.71 ± 0.13	0.47 (0.24,0.70)	15.599	0.000^**^	3.43 ± 0.09	−0.18 (−0.39,0.06)	2.14	0.14	−0.72 (−1.03,-0.41)	0.000^**^
OSA	Baseline	2.67 ± 0.18				2.86 ± 0.16				−0.19 (−0.66,0.29)	0.448
	Immediately post-test	2.47 ± 0.16	0.20 (−0.20,0.59)	0.947	0.33	2.94 ± 0.15	−0.09 (−0.42,0.24)	0.281	0.60	−0.47 (−0.90,-0.05)	0.015^*^
	1-month post-test	2.29 ± 0.12	0.37 (0.01,0.73)	3.952	0.047^*^	3.02 ± 0.14	−0.16 (−0.46,0.14)	1.085	0.30	−0.72 (−1.08,-0.36)	0.000^**^

AMIS, addiction memory intensity scale; VC, visual clarity; OSA, other sensory aspects. CI, confidence interval, ^*^p < 0.05, ^**^p < 0.01.

### 3.4. Psychological craving

The results of the GEE showed that: (1) the main effect of group was significant (Wald*χ*^2^ = 7.063, *p* = 0.008, Partial η^2^ = 0.105); (2) the main effect of time was significant (Wald*χ*^2^ = 40.026, *p* < 0.000, Partial η^2^ = 0.356); (3) and the group ×time interaction effect was significant (Wald*χ*^2^ = 27.832, *p* < 0.0001, Partial η^2^ = 0.278).

At baseline, there was no significant difference in VAS score between the intervention and control groups (36.72 ± 3.13 vs. 30.17 ± 4.14, *p* = 0.222). The VAS score was significantly lower in the intervention than in the control group, both immediately after the intervention (7.59 ± 1.65 vs. 27.67 ± 3.99, *p* < 0.0001) and 1 month thereafter (10.17 ± 2.42 vs. 27.67 ± 3.58, *p* < 0.0001).

After the intervention, the VAS score decreased significantly (7.59 ± 1.65 vs. 36.72 ± 3.13, *p* < 0.0001) and remained low after 1 month (36.72 ± 3.13 vs. 10.17 ± 2.42, *p* < 0.0001). There was no significant difference in VAS score among the three test times in the control group (*p* > 0.05).

### 3.5. Physiological parameters

#### 3.5.1. Heart rate difference

The results of the GEE showed that: (1) the main effect of group was significant (Wald*χ*^2^ = 10.918, *p* = 0.001, Partial η^2^ = 0.195); (2) the main effect of time was not significant (Wald*χ*^2^ = 5.232, *p* = 0.073, Partial η^2^ = 0.039); (3) and the group × time interaction effect was significant (Waldχ^2^ = 19.820, *p* < 0.0001, Partial η^2^ = 0.114).

At baseline, there was no significant difference in heart rate difference between the intervention and control groups (5.07 ± 1.30 vs. 5.23 ± 1.04, *p* = 0.921). The difference value in heart rate was significantly lower in the intervention than control group, both immediately (1.21 ± 0.37 vs. 6.47 ± 0.92, *p* < 0.0001) and 1 month after the end of the intervention (1.45 ± 0.36 vs. 6.36 ± 0.76, *p* < 0.0001).

In the intervention group, the difference value in heart rate was reduced significantly after the intervention compared to baseline (1.21 ± 0.37 vs. 5.07 ± 1.30, *p* < 0.0001), and remained low after 1 month (1.45 ± 0.36 vs. 5.07 ± 1.30，*p* = 0.002). In the control group, the heart rate difference was significantly higher at posttest than baseline (6.47 ± 0.92 vs. 5.23 ± 1.04, *p* = 0.002) and was not significantly different from baseline at 1 month after the intervention (6.36 ± 0.76 vs. 5.23 ± 1.04, *p* = 0.11).

#### 3.5.2. Diastolic blood pressure difference

The results of the GEE showed that: (1) the main effect of group was not significant (Wald*χ*^2^ = 3.067, *p* = 0.08, Partial η^2^ = 0.064); (2) the main effect of time was significant (Wald*χ*^2^ = 10.1, *p* = 0.006, Partial η^2^ = 0.067); and (3) the group × time interaction effect was significant (Wald*χ*^2^ = 8.104, *p* = 0.017, Partial η^2^ = 0.051).

At baseline, there was no significant difference in diastolic blood pressure difference between the intervention and control groups (9.17 ± 2.32 vs. 8.53 ± 1.66, *p* = 0.823). The diastolic blood pressure difference was significantly lower in the intervention than control group, both immediately (2.66 ± 0.85 vs. 8.20 ± 1.49, *p* = 0.001) and 1 month after the end of the intervention (3.17 ± 0.89 vs. 8.20 ± 1.49, *p* = 0.007).

After the intervention, the difference value in diastolic blood pressure decreased significantly (2.66 ± 0.85 vs. 9.17 ± 2.32, *p* = 0.002) and remained low after 1 month (3.17 ± 0.89 vs. 9.17 ± 2.32, *p* = 0.006). There was no significant difference in diastolic blood pressure difference among the three test times in the control group (*p* > 0.05).

#### 3.5.3. Systolic blood pressure difference

The results of the GEE showed that: (1) the main effect of group was significant (Wald*χ*^2^ = 4.503, *p* = 0.034, Partial η^2^ = 0.078); (2) the main effect of time was not significant (Wald*χ*^2^ = 4.231, *p* = 0.121, Partial η^2^ = 0.037); and (3) the group × time interaction effect was not significant (Wald*χ*^2^ = 5.558, *p* = 0.062, Partial η^2^ = 0.024).

There was no significant difference in the systolic blood pressure difference between the intervention and control groups at baseline (7.21 ± 2.44 vs. 7.66 ± 1.66, *p* = 0.876). The systolic blood pressure difference was significantly lower in the intervention than control group, both immediately (2.17 ± 0.61 vs. 9.50 ± 2.44, *p* = 0.004) and 1 month after the end of the intervention (2.31 ± 1.10 vs. 6.83 ± 1.53, *p* = 0.016).

In the intervention group, the systolic blood pressure difference at the posttest decreased significantly compared to baseline (2.17 ± 0.61 vs. 7.21 ± 2.44, *p* = 0.025). After 1 month it remained below the baseline value (2.31 ± 1.10 vs. 7.21 ± 2.44, *p* = 0.052), but not significantly. There was no significant difference in systolic blood pressure difference among the three test times in the control group (*p* > 0.05).

### 3.6. Addiction memory intensity

#### 3.6.1. Addiction memory intensity score

The results of the GEE showed that: (1) the main effect of group was significant (Wald*χ*^2^ = 7.009, *p* = 0.008, Partial η^2^ = 0.107); (2) the main effect of time was significant (Wald*χ*^2^ = 9.703, *p* = 0.008, Partial η^2^ = 0.047); (3) and the group ×time interaction effect was significant (Wald*χ*^2^ = 15.559, *p* < 0.0001, Partial η^2^ = 0.097).

At baseline, there was no significant difference in AMIS score between the intervention and control groups (3.02 ± 0.16 vs. 3.13 ± 0.12, *p* = 0.586). The AMIS score was significantly lower in the intervention than in the control group, both immediately (2.81 ± 0.14 vs. 3.25 ± 0.10, *p* = 0.037) and 1 month after the end of the intervention (2.55 ± 0.11 vs. 3.22 ± 0.12, *p* < 0.0001).

In the intervention group, the AMIS score was reduced after the intervention compared to baseline, but there was no significant difference (2.81 ± 0.14 vs. 3.02 ± 0.16，*p* = 0.19). However, 1 month after the intervention, the AMIS score was significantly lower than that at baseline (2.55 ± 0.11 vs. 3.02 ± 0.16, *p* < 0.0001). There were no significant differences among the three test times in the control group (*p* > 0.05).

#### 3.6.2. Visual clarity score

The results of the GEE showed that: (1) the main effect of group was significant (Wald*χ*^2^ = 7.038, *p* = 0.008, Partial η^2^ = 0.108); (2) the main effect of time was not significant (Wald*χ*^2^ = 5.697, *p* = 0.058, Partial η^2^ = 0.028); and (3) the group ×time interaction effect was significant (Wald*χ*^2^ = 18.008, *p* < 0.0001, Partial η^2^ = 0.103).

At baseline, there was no significant difference in visual acuity score between the intervention and control groups (3.17 ± 0.17 vs. 3.26 ± 0.12, *p* = 0.678). The visual clarity score was significantly lower in the intervention than in the control group, both immediately (2.97 ± 0.16 vs. 3.40 ± 0.10, *p* = 0.027) and 1 month after the end of the intervention (2.71 ± 0.13 vs. 3.43 ± 0.09, *p* < 0.0001).

In the intervention group, the visual clarity score at the posttest decreased compared to baseline, but not significantly (2.97 ± 0.16 vs. 3.17 ± 0.17, *p* = 0.260). However visual clarity 1 month after the intervention was significantly lower than at baseline (2.71 ± 0.13 vs. 3.17 ± 0.17, *p* < 0.0001). There was no significant difference in visual clarity among the three test times in the control group (*p* > 0.05).

#### 3.6.3. Other sensory aspects score

The results of GEE showed that: (1) the main effect of group was significant (Wald*χ*^2^ = 7.079, *p* = 0.008, Partial η^2^ = 0.107); (2) the main effect of time was not significant (Wald*χ*^2^ = 1.012, *p* = 0.603, Partial η^2^ = 0.007); and (3) the group ×time interaction effect was significant (Wald*χ*^2^ = 6.191, *p* = 0.045, Partial η^2^ = 0.046).

There was no significant difference in the intensity of other sensory aspects between the intervention and control groups at baseline (2.67 ± 0.18 vs. 2.86 ± 0.16，*p* = 0.448). The intensity of other sensory aspects was significantly lower in the intervention than in the control group, both immediately (2.47 ± 0.16 vs. 2.94 ± 0.15, *p* = 0.015) and 1 month after the end of the intervention (2.29 ± 0.12 vs. 3.02 ± 0.14, *p* < 0.0001).

In the intervention group, the intensity of other sensory aspects at the posttest decreased compared to baseline, but not significantly (2.47 ± 0.16 vs. 2.67 ± 0.18, *p* = 0.330). However, the intensity of other sensory aspects 1 month after the intervention was significantly lower than at baseline (2.29 ± 0.12 vs. 2.67 ± 0.18, *p* < 0.0001). There was no significant difference in the intensity of other sensory aspects among the three test times in the control group (*p* > 0.05).

## 4. Discussion

This study used VR technology, combined with motivational reinforcement therapy and addiction memory reconsolidation theory, to design an intervention program aimed at reducing psychological craving and decreasing the intensity of addiction memories in MA-dependent female young adults. This is the first intervention program to be implemented in an MA-dependent female adolescent population. Furthermore, we evaluated its effectiveness based on memory strength, psychological craving and physiological response. The results showed that, after the intervention, psychological craving and the difference values of physiological parameters significantly decreased and remained at a low level for 1 month. However, patients who did not receive the intervention showed no significant change in psychological craving or physiological response. Meanwhile, the intervention effectively reduced the addiction memory intensity of the MA-dependent female young adults, and a consistent decrease in addiction memory intensity, visual clarity, and the intensity of other sensory aspects were seen over time. These results suggest that the VR-based motivation enhancement+desensitization treatment is effective for reducing the psychological craving of MA-dependent female young adults and reducing the intensity of their addiction memories.

According to memory reconsolidation theory, researchers extracted addicts’ original addiction memories, activated them to induce an unstable state, and then intervened within a specific time window (10 min ~ 6 h) to change or eliminate the original memory connections (20). The key to this process is that the original memory is activated to induce an unstable state (21). In this study, addictive memories were activated to an unstable state by exposure to meth-related cues in a virtual reality environment, followed immediately by relaxation exercises to interfere with the memory reconsolidation process during an effective time window, thereby abating addictive memory and reducing psychological craving. Female MA-dependent young adults experienced a significant increase in heart rate and blood pressure upon entering the meth-related virtual reality environment, suggesting that immersion in the MA-related virtual reality environment successfully elicited a physiological response in MA-dependent individuals. This result is similar to that of a study conducted with cocaine-dependent patients (22). After cocaine-dependent patients entered the cocaine-related virtual reality environment, the patients’ subjective emotional responses, heart rate and electrodermal indicators showed that the stimulus-rich and standardised virtual reality scenario was effective in eliciting subjective psychological craving and physiological response. This was a key point in making the intervention effective, indirectly reflecting the effectiveness of the manipulation in activating addiction memories and inducing psychological craving. The use of VR to construct drug-related scenarios has higher ecological validity, is more realistic, and can be used to present composite cues that help activate addiction memories and facilitate the goal of eliminating or changing addiction memories. The effectiveness of an intervention comprising VR combined with memory reconsolidation was demonstrated by Maples-Keller et al. (23). The physiological response to fear was effectively suppressed in patients treated by the VR combined with memory reconsolidation intervention. Additionally, VR is advantageous for inducing craving (24), which can enhance the effect of extinction interventions to better achieve craving reduction. Studies have reported significant reductions in nicotine and alcohol craving in addicts using VR interventions (25–29). Some researchers have also combined VR with extinction interventions and cognitive behavioral therapy for nicotine addicts, and reported a decrease in subjective cravings and reduced smoking behavior (30). Liu et al. confirmed the effectiveness of VR combined with cue exposure for memory extinction in MA-dependent patients (31). The training attenuated the patients’ craving for drugs and responsivity to cues. Interventions for addiction memory and psychological craving in the VR environment have important implications for relapse prevention. Although the patient’s symptoms can be improved in a therapeutic environment, the most important change is not in the laboratory or during treatment, but rather in the world in which the patient lives (32). The advantage of VR for addiction interventions is that it provides a variety of environments that resemble real-life scenarios, including meth-dependent persons, thus allowing for better transfer of intervention effects to real life. Our study used VR to present drug cues that effectively activated addiction memories and induced psychological craving. Our results are similar to previous studies that used craving to assess a VR-based craving-abatement intervention. This intervention protocol was effective for reducing craving in MA-dependent female young adults. Furthermore, the results of this study provide direct evidence that this intervention protocol can reduce addiction memory intensity, visual clarity, and sensory intensity in MA-dependent female young adults. More notably, the effect of the intervention persisted for 1 month.

In this study, we included a one-on-one interview and motivational reinforcement phases before the formal desensitization training, which also played a key role in the efficacy of the motivational interview. According to the motivation-based integrative theory of addiction, motivational interviewing is an individualized, comprehensive treatment technique that stimulates internal motivation for, and guides, sustained behavior changes (33). In the one-on-one motivational interview phase of our intervention, the therapist established a good therapeutic alliance with the patient. During this phase, the therapist stimulated the patient’s internal drive for sustained change, which was consolidated during the subsequent desensitization training. In the motivational reinforcement phase, the patient’s motivation to quit was further enhanced. Through group discussions about the consequences of addiction, expectations of recovery, and the meaning of life, therapists helped their patients probe inner conflicts associated with their addictive behaviors more deeply. Through this process, patients accrue important resources to help them maintain long-term detoxification. Every time a memory is extracted, we automatically process it according to the present-day context, after which the modified memory replaces the original one and is stored in long-term memory (34). By providing the VR-based desensitization training after the motivational reinforcement phase, the patients are motivated during the memory extraction-reconsolidation process. Thus, during the memory reconsolidation phase, not only has the present-day context changed, but also its psychological context relative to the original memory. Wang evaluated the effects of a “motivation enhancement-desensitization-neurotransmitter regulation” intervention in patients with MA dependence. The results showed that an intervention model combining motivational reinforcement and desensitization increased patients’ motivation to detoxify and reduced the intensity of their addiction memories (12). Our findings also suggest that motivational reinforcement prior to desensitization training is effective for reducing the intensity of addiction memories and psychological craving in MA-dependent female young adults.

The present study also had some limitations. First, it was an exploratory intervention that only included female MA-dependent young adults, which limits the generalizability of the results. In the future, we plan to conduct a multicenter, large-scale study to validate the efficacy of this protocol in a larger group, and further analyze and explore specific intervention mechanisms. Second, although the intervention in this study had a specific operational procedure, the results may have been biased to some degree due to its psychotherapeutic nature, which precluded blinding of the subjects. Finally, although the results of this study demonstrate the effectiveness of the intervention for reducing the intensity of addiction memory and psychological craving 1 month after the end of the intervention, further follow-up studies are needed to determine the long-term effects of the intervention.

In conclusion, this study first combined VR technology with a memory reconsolidation intervention, and added an element of motivational reinforcement, to devise a novel protocol for intervening in psychological craving and addiction memory; the results were highly promising. This study not only promotes the development and application of memory reconsolidation-based clinical treatments and interventions for addiction, but also provides new evidence that could aid the further development of addiction treatment theory.

## Data availability statement

The original contributions presented in the study are included in the article/supplementary material, further inquiries can be directed to the corresponding author.

## Ethics statement

The studies involving human participants were reviewed and approved by Chengdu Medical College Biomedical Ethics Committee. The patients/participants provided their written informed consent to participate in this study.

## Author contributions

XJ and LJ designed the experiment XJ performed it. YT analyzed the experimental data and participated in the paper writing. XJ drafted the manuscript and prepared the published works. LJ reviewed and edited the manuscript. LZ provided the experimental equipment. BW, YD, SZ, and YY assisted in recruiting subjects, and participated in the implementation of the experiment. All authors contributed to the article and approved the submitted version.

## Funding

This work was supported by Key R&D Project of Sichuan Provincial Department of Science and Technology (Item Number: 2020YFS0348), Graduate Student Innovation Fund Key Project (Item Number: YCX2021-01).

## Conflict of interest

The authors declare that the research was conducted in the absence of any commercial or financial relationships that could be construed as a potential conflict of interest.

## Publisher’s note

All claims expressed in this article are solely those of the authors and do not necessarily represent those of their affiliated organizations, or those of the publisher, the editors and the reviewers. Any product that may be evaluated in this article, or claim that may be made by its manufacturer, is not guaranteed or endorsed by the publisher.
